# Transmission of multidrug-resistant tuberculosis in Shimen community in Shanghai, China: a molecular epidemiology study

**DOI:** 10.1186/s12879-021-06725-0

**Published:** 2021-10-29

**Authors:** Zhiying Han, Jing Li, Guomei Sun, Kaikan Gu, Yangyi Zhang, Hui Yao, Yuan Jiang

**Affiliations:** 1 Department of Tuberculosis Prevention and Control, Jing’an District Center for Disease Control and Prevention, Shanghai, 200072 China; 2 Tuberculosis Laboratory, Shanghai Municipal Center for Disease Control and Prevention, Shanghai, 200036 China; 3 Second Shimen Road Community Health Center, Shanghai, China

**Keywords:** Transmission, Molecular epidemiology, Multidrug-resistant tuberculosis

## Abstract

**Background:**

Multidrug-resistant tuberculosis (MDR-TB) has become a major public health problem in China, with mounting evidence suggesting that recent transmission accounts for the majority of MDR-TB. Here we aimed to reveal the transmission pattern of an MDR-TB outbreak in the Jing'an District of Shanghai between 2010 and 2015.

**Methods:**

We used whole-genome sequencing (WGS) to conduct genomic clustering analysis along with field epidemiological investigation to determine the transmission pattern and drug resistance profile of a cluster with ten MDR-TB patients in combining field epidemiological investigation.

**Results:**

The ten MDR-TB patients with genotypically clustered Beijing lineage strains lived in a densely populated, old alley with direct or indirect contact history. The analysis of genomic data showed that the genetic distances of the ten strains (excluding drug-resistant mutations) were 0–20 single nucleotide polymorphisms (SNPs), with an average distance of 9 SNPs, suggesting that the ten MDR-TB patients were infected and developed the onset of illness by the recent transmission of *M. tuberculosis*. The genetic analysis confirmed definite epidemiological links between the clustered cases.

**Conclusions:**

The integration of the genotyping tool in routine tuberculosis surveillance can play a substantial role in the detection of MDR-TB transmission events. The leverage of genomic analysis in combination with the epidemiological investigation could further elucidate transmission patterns. Whole-genome sequencing could be integrated into intensive case-finding strategies to identify missed cases of MDR-TB and strengthen efforts to interrupt transmission.

**Supplementary Information:**

The online version contains supplementary material available at 10.1186/s12879-021-06725-0.

## Introduction

According to the global tuberculosis report [1], there are an estimated 465,000 rifampicin-resistant tuberculosis (RR-TB) patients in 2019 worldwide, of which about 78% were multidrug-resistant tuberculosis (MDR-TB). China has the second-highest burden of MDR-TB and has approximately 65,000 incident cases of RR-TB in 2019 in the Chinese mainland, accounts for 14% of RR-TB worldwide [1]. According to the TB Information Management System in Shanghai, 3395 TB cases were reported in 2019 with a reported incidence of 25/100,000, with an overall drug-resistance rate of 11.7% and MDR-TB of 3.7%. Understanding the transmission pattern of MDR-TB is of great importance to prevent and control TB.

Due to the remarkable variability in the timing of disease progression after infection, it was difficult to measure and track the transmission of TB. The application of genotyping techniques of *Mycobacterium tuberculosis* (*M. tuberculosis*) provides a useful tool for researchers to explore the transmission patterns of TB and the risk factors associated with transmission [2]. Previous methods used for *M. tuberculosis* molecular epidemiology that have provided useful insight to understand transmission and differentiate between endogenous onset and exogenous reinfection of drug-resistant strains include insertion sequence 6110-restriction fragment length polymorphism (IS6110), spoligotyping, and variable numbers of tandem repeats (VNTR) techniques [3, 4]. Over time, the resolution of the genotyping tool has been ultimately improved as the development of high-throughput whole-genome sequencing (WGS) in the studies of molecular epidemiology of TB transmission. The WGS can confirm the variation of genetic diversity among clustered strains to construct a relatively accurate transmission chain may provide insight into source cases and potential missing cases, an advantage compared to the traditional epidemiological investigation and genotyping methods. [5–8]

A recent study has found that MDR-TB cases in Shanghai were mostly attributed to direct transmission of drug-resistant strains in Shanghai, and part of the clustered strains was found in the Jing'an District [8]. Previously, to study transmission patterns of TB in this district, we conducted a molecular epidemiology study with the clinical *M. tuberculosis* isolates from patients diagnosed in Jing'an Designated Hospital from 2010 to 2015. By using the "9 + 3" VNTR genotyping loci set [9], we identified genotypically clustered strains. The largest identified cluster included ten MDR-TB cases, of which all the patients were living in the same community and had symptom onset over ten years. Here, we performed combined whole-genome sequencing analysis and field epidemiological investigation of these ten MDR-TB patients, to further understand the transmission pattern and the cause of this MDR-TB cluster. The result of this study could provide evidence to inform more efficient MDR-TB control strategies in urban areas.

## Methods

### Data source

At Jing’an Designated Hospital from 2010–2015, one hundred seventy-three pulmonary TB patients were diagnosed, of which 92 cases were culture positive. From the Shanghai Municipal Center for Disease Control and Prevention (Shanghai CDC), we collected 80 of 92 effective strain samples in this district for genotyping analysis. All of the *M. tuberculosis* isolates were sent to the reference laboratory for drug susceptibility testing (DST) using the proportion method on Lowenstein-Jensen medium at the following concentrations: rifampicin (RIF) 40 µg/mL, isoniazid (INH) 0.2 µg/mL, ethambutol (EMB) 2.0 µg/mL, and streptomycin (SM) 4.0 µg/mL. As described previously, we conducted VNTR genotyping for all strains using the 9 + 3 loci set with hypervariable loci that were developed specifically for discriminating of *M. tuberculosis* strains in China and identified 65 genotypes, with 22 strains forming 7 clusters. [9, 10] The largest cluster of ten MDR-TB patients was selected for further analysis in this study.

#### Whole-genome sequencing, clustering analysis, and transmission inference

We performed whole-genome sequencing analysis on MDR-TB strains from all ten patients. The genomic DNAs were extracted following the cetyl trimethyl ammonium bromide (CTAB) method. [11] The reference laboratory and data center at the Shanghai CDC performed the DNA library preparation and whole-genome sequencing using Illumina Hiseq 2500 with an average coverage of 137 times with a range from 112 to 180 times. Analysis of the raw sequencing data was completed using the standard process pipeline according to previous studies. [12] High quality and trimmed sequencing reads were mapped to H37Rv reference sequence (NC_000962.3) using Bowtie2 (v2.2.9). [13] We used Samtools (v1.6) together with VarScan (v2.3.9) to call the single-nucleotide polymorphisms (SNPs) and identify fixed genomic variants (frequency ≥ 75%). [14][14]. The fixed SNPs, excluding those in the region of PE-PGRS and PE-PPE genes, and drug-resistance associated genes, were combined into a concatenated alignment. We then used this alignment to construct a maximum-likelihood phylogeny using MEGA (version 9.0) with a general time-reversible (GTR) substitution model and 1,000 bootstraps (Additional file 1: Figure S1 and S2, appendix). We defined genomic clusters as those strains within a threshold of twelve or fewer SNPs [8]. We determined the sublineage and drug-resistant gene mutations based on the WGS data analysis by using an in-house Perl script and TB-Profiler (v2.8.14). We also used the SpoTyping (version 2.0) to generate the spoligotyping pattern from the sequenced data [16].

We used a Bayesian evolutionary analysis to infer a timed phylogeny by sampling trees with BEAST (version 1.8.4) by using the concatenated alignment and labeled the time tips of each strain using the diagnosed date. We used the GTR substitution model with a coalescent constant population size and a strict molecular clock rate. The input XML file was modified to specify the number of invariant sites. The model was run using a Markov Chain Monte Carlo (MCMC) chain length of 10,000,000 with a 10% burn-in. A maximum clade credibility tree was generated using TreeAnnotator (v2.4.7) and visualized using FigTree (v1.4.2). This timed phylogeny tree was then used as input for the transmission tree inference using the R package called TransPhylo and followed the previously described methods. [17] We used a Gamma distribution with a shape parameter of 1.3 and a rate parameter of 0.3 for the generation time density, with consideration of the generation time of tuberculosis that allowed a rapid progression to active disease after infection, but also the possibility of a long latent period before active progression. [17]

#### Epidemiological investigation

Epidemiological investigations were used to identify the persons, places, and behaviors associated with the transmission of *M. tuberculosis* between TB patients in this cluster. A standard questionnaire was used to collect information about the individual's place of residence, close contacts, interpersonal relationships, frequently visited locations, and the situation of hospital visits as previously described for this cluster [8]. Epidemiological links were defined as follows: "confirmed epidemiological links" were recognized if two patients were close contacts/acquaintances or resided in the same location; "probable epidemiological links" were recognized if two patients resided in a neighborhood or frequently shared common spaces.

All interviews were performed retrospectively by trained interviewers after obtaining informed consent. If the patient was not able to participate in the interview themselves, close contact of the patient (e.g., spouse, children, parents) was interviewed. On-site investigation of TB patients was carried out according to national TB control guidelines.

## Results

### Demographic features and TB history

Among the ten MDR-TB patients included, eight were male. Six patients had no previous history of pulmonary TB. Five of ten were retired persons, and another five were unemployed. The mean age was 56 years, with a range from 35 to 75. (Table 1). Six patients had no history of antituberculous therapy, of which four patients were seriously ill. Of the four remaining patients with a previous history of treatment, three had severe disease. Half of the ten MDR-TB patients died and three of them were attributed to TB disease. One was discontinued due to drug side effects, and two were still in the period of follow-up (interrupted treatment due to severe side effects). Only one patient was cured. (Table 1).

**Table 1:** Characteristics of multidrug-resistant tuberculosis in the cluster (n = 10)

Patient ID	Sex	Age, years	Previous treatment	Onset/diagnosed time (DD-MM-YY)	Treatment outcomes	Relationship	Nature of epidemiological link
2010–0183	Male	49	No	10-Sep-09 15-Nov-09	Died	Couple	All lived in the same residential community or alley; Neighborhood; Game room or restaurant within the alley
2012–1261	Female	35	No	11-May-12 23-May-12	Still on treatment*		
2010–1007	Male	75	No	10-Jul-09 21-Oct-09	Interrupted	Father and son	
2016–0569	Male	58	Yes	03-Jan-15 26-Jan-15	Still on treatment*		
2012–0659	Female	52	Yes	01-Mar-06 21-Jul-11	Died	Couple	
2012–1614	Male	58	Yes	02-Apr-06 18-Sep-12	Died		
2013–0285	Male	53	No	25-Dec-12 10-Jan-13	Cured	Neighborhood	
2012–1050	Male	56	No	01-Mar-12 13-Mar-12	Died		
2012–1220	Male	63	Yes	01-Mar-09 17-Jan-12	Died	ND	
2010–1008	Male	61	No	28-Mar-10 06-May-10	Treatment completed	ND	Casually visit the game room

*Patients 2012–1261 and 2016–0569 were still on treatment when the investigation was performed

### Epidemiological investigation

Among the ten patients, nine had confirmed epidemiological links, including two couples (patient ID: 2010–0183 and 2012–1261; 2012–0659 and 2012–1614) and one father-and son-pair (2010–1007 and 2016–0569). These nine patients were acquaintances: 8 resided in the same alley, the 9th patient (2012–1220) lived on the ground floor of another alley within 200 m, and owned a fast-food restaurant. The remaining patient had "probable epidemiological links" as defined above, and lived in an alley about two kilometers away from the address of 9 other patients (Table 1). Eight of the ten patients who visited the same game room frequently in this ally.

Table 2 shows the dates of symptom onset, TB diagnosis, treatment course, and hospitalization. Both patients 2012–0659 and 2012–1614, who belonged to a couple, experience symptom onset in 2006. They experienced long delays and interrupted treatment from 2006 to 2011. The remaining eight patients experienced a median diagnosis delay of 15 days of seeking health care (IQR, 12–65 days). Although ten patients visited the same hospital to follow up, only two of the ten patients had an overlapping hospital stay for a few days during the middle of their hospitalization. (Table 2).

**Table 2:**
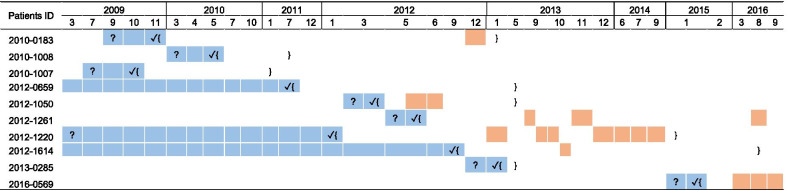
The time (by year and month) of onset of symptoms, confirmation of diagnosis, treatment course and duration of hospitalization of the ten MDR-TB cases

? = onset of TB like symptoms (*Patient 2012–0659 and 2012–1614 had the TB symptom back to the time of 2003); ✓ = confirmation of diagnosis; {} represents the treatment course, patients 2016–0569 and 2012–1261 were still on treatment; the cells in blue represent the delay of diagnosis and the cells in orange represents hospitalization

### Sublineage, drug resistance mutations, and phylogenetic analysis

All ten MDR-TB patients were infected with strains belonging to the Beijing family in sublineage 2.2.2 (Modern Beijing strain), and had the same spologotyping pattern (000000000003771). Table 3 showed the drug susceptibility profiles and drug resistance mutations in related genes. Each of the ten patients had the same fixed INH, RIF, and SM resistant related gene mutations (*katG*-315S/T, *rpoB*-450S/L, and *rpsL*-43 K/R respectively). The other DR mutations were described below in the transmission chain analysis. We did not observe any fixed drug-resistant mutations to second-line anti-TB drugs, including amikacin, kanamycin, and capreomycin.

**Table 3:** Profile of phenotypic resistance and drug-resistant related gene mutants of the MDR-TB cases

Patient ID	Phenotypic resistance (SIRE)	INH	RIF	SM	EMB	PZA	FLQ	Am	Cm	Km
2010–1007	R-R-R-R	*katG-*S315T	*rpoB-*S450L	*rpsL-*K43R	*embB*-M306V	*pncA*-V155G	*gyrA*-A90V	–	–	–
2010–1008	R-R-R-R	*katG-*S315T	*rpoB-*S450L	*rpsL-*K43R	*embB*-M306V	*pncA*-V155G	*gyrA*-A90V	–	–	–
2010–0183	R-R-R-R	*katG-*S315T	*rpoB-*S450L	*rpsL-*K43R	*embB*-M306V	*pncA*-V155G	–	–	–	–
2012–1050	R-R-R-R	*katG-*S315T	*rpoB-*S450L	*rpsL-*K43R	*embB*-M306V	*pncA*-V155G	–	–	–	–
2012–1220	R-R-R-R	*katG-*S315T	*rpoB-*S450L	*rpsL-*K43R	*embB*-M306V	*pncA*-V155G	*gyrA*-D94G	–	–	–
2012–1261	R-R-R-R	*katG-*S315T	*rpoB-*S450L	*rpsL-*K43R	*embB*-M306V	*pncA*-T47P	–	–	–	–
2012–0659	R-R-R-R	*katG-*S315T	*rpoB-*S450L	*rpsL-*K43R	*embB*-M306V	*pncA*-C14Y	*gyrA*-D94A	–	–	–
2013–0285	R-R-R- R	*katG-*S315T	*rpoB-*S450L	*rpsL-*K43R	*embB*-M306V	*pncA*-V155G	-	–	–	–
2016–0569	R-R-R-S	*katG-*S315T	*rpoB-*S450L	*rpsL-*K43R	–	–	–	–	–	–
2012–1614	R-R-R-S	*katG-*S315T	*rpoB-*S450L	*rpsL-*K43R	–	–	–	–	–	–

*R* resistant, *S* susceptible, *INH* isoniazid, *RIF* rifampicin, *EMB* ethambutol, *PZA* pyrazinamide, *SM* streptomyces *FLQ* fluoroquinolones, *Am* amikacin, *Km* kanamycin, *Cm* capreomycin

**Fig. 1:**
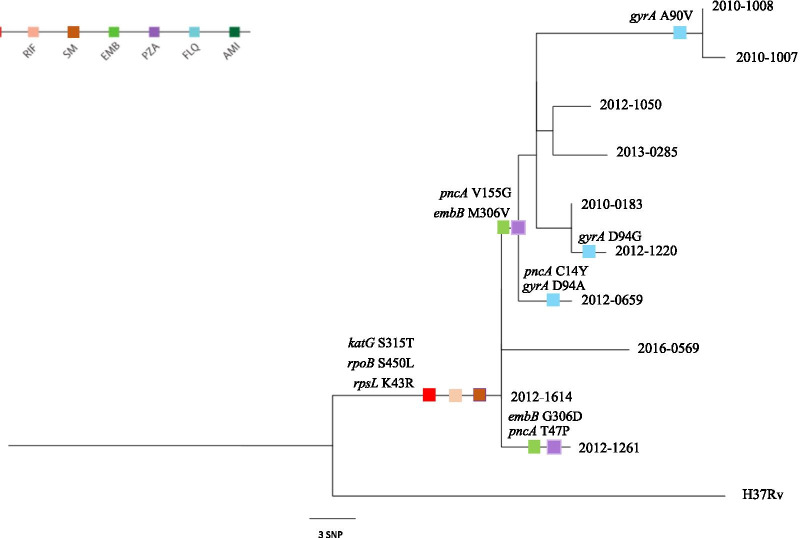
Maximum-likelihood, single-nucleotide polymorphism-based phylogeny of multidrug-resistant isolates and mutations of drug-resistant genes among ten clustered multidrug-resistant TB strains. This phylogenetic tree was based on 10 sequences with a total 68 core single nucleotide variant positions (MEGA v9.0)

WGS showed that the genomic differences between any two strains (excluding drug-resistant mutations) ranged from 0 to 20 SNPs, and the average difference was 9.1 SNPs (Table 4), consistent with the VNTR genotyping analysis and supporting recent transmission. We built a maximum-likelihood (M-L) phylogenetic tree based on the SNPs alignment (Fig. 1) and mapped the drug resistance mutations to the M-L tree. The accumulation of drug-resistant mutations, along with the drug-susceptibility testing, suggest that this cluster arose from the transmission of MDR *M. tuberculosis* strain.

**Table 4:**
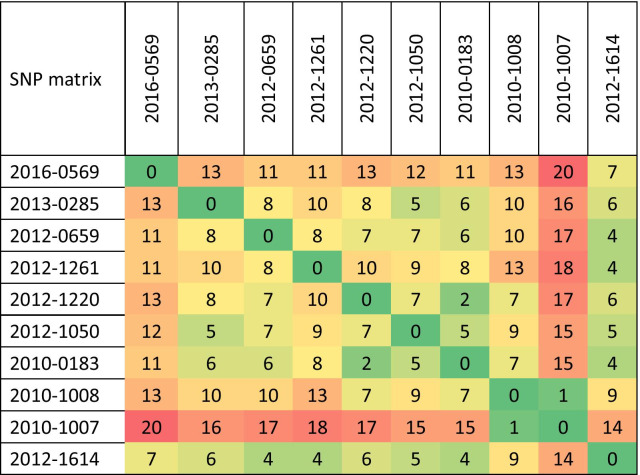
Pairwise SNPs distance between the 10 MDR-TB strains in the cluster

### Transmission chain inference

Additiona file 1: Figure S3 shows the transmission inference based on the timed phylogeny tree analysis. The putative transmission source or index case was initialed before 2006, which was in consistent with the investigation that patients 2012–1614 and 2012–0659, who were a couple, had presented symptoms in March and April 2006, respectively. Both strains from this couple were in the root of the M-L tree in Fig. 1, indicating the putative early transmission events related to these two patients.

Based on the alignment of the SNPs of the ten MDR *M. tuberculosis* strains, we mapped the drug-resistant mutations on the phylogenetic tree of each node (Fig. 1). We observed consistent mutation profiles of drug resistance genes of INH, RFP, and SM for each of the ten strains. Meanwhile, those strains located at the end branch had several unique drug-resistance mutations, including the *gyrA* A90C in 2010–1007 and 2010–1008, and *gyrA* D94G in 2012–1220. These findings provide further evidence of direct transmission of MDR-TB strains and suggest the acquisition of additional drug-resistance mutation during the transmission of MDR-TB strains.

The timed-tree in Additional file 1: Figure S3 also suggests at least two transmission routes initialed by the source case, one subsequentially transmitted to the four individuals, including 2012–1261, 2016–0569, and the patients in a couple 2012–1614 and 2012–0659. This finding was supported by the relatively small genetic differences (less than four SNPs) between the patients 2012–1614 (husband of patient 2012–0659), 2012–1261 (living in the same alley), and 2010–0183 (living in the same alley) (Table 4). Another route includes several transmission events to the remaining six cases. The shared mutation of both ethambutol (EMB) and pyrazinamide (PZA) genes among these six patients (Table 3) suggest that they may be transmitted from a common source case.

## Discussion

Based on a genotyping analysis of *M. tuberculosis* in a district in Shanghai, we conducted a genomic and epidemiological analysis of ten MDR-TB strain cluster and described the putative transmission chain of this cluster. With the development of next-generation high-throughput sequencing technology, whole-genome sequencing analysis has been gradually applied to the molecular epidemiological studies of *M. tuberculosis* [7, 18, 19], which can determine the variation and transmission source in a transmission chain [20]. Because the reverse mutation of single nucleotide polymorphism (SNP) is rare [21, 22], WGS data can accurately describe transmission direction by comparing differences in SNPs between TB strains in a cluster.

**Fig. 2:**
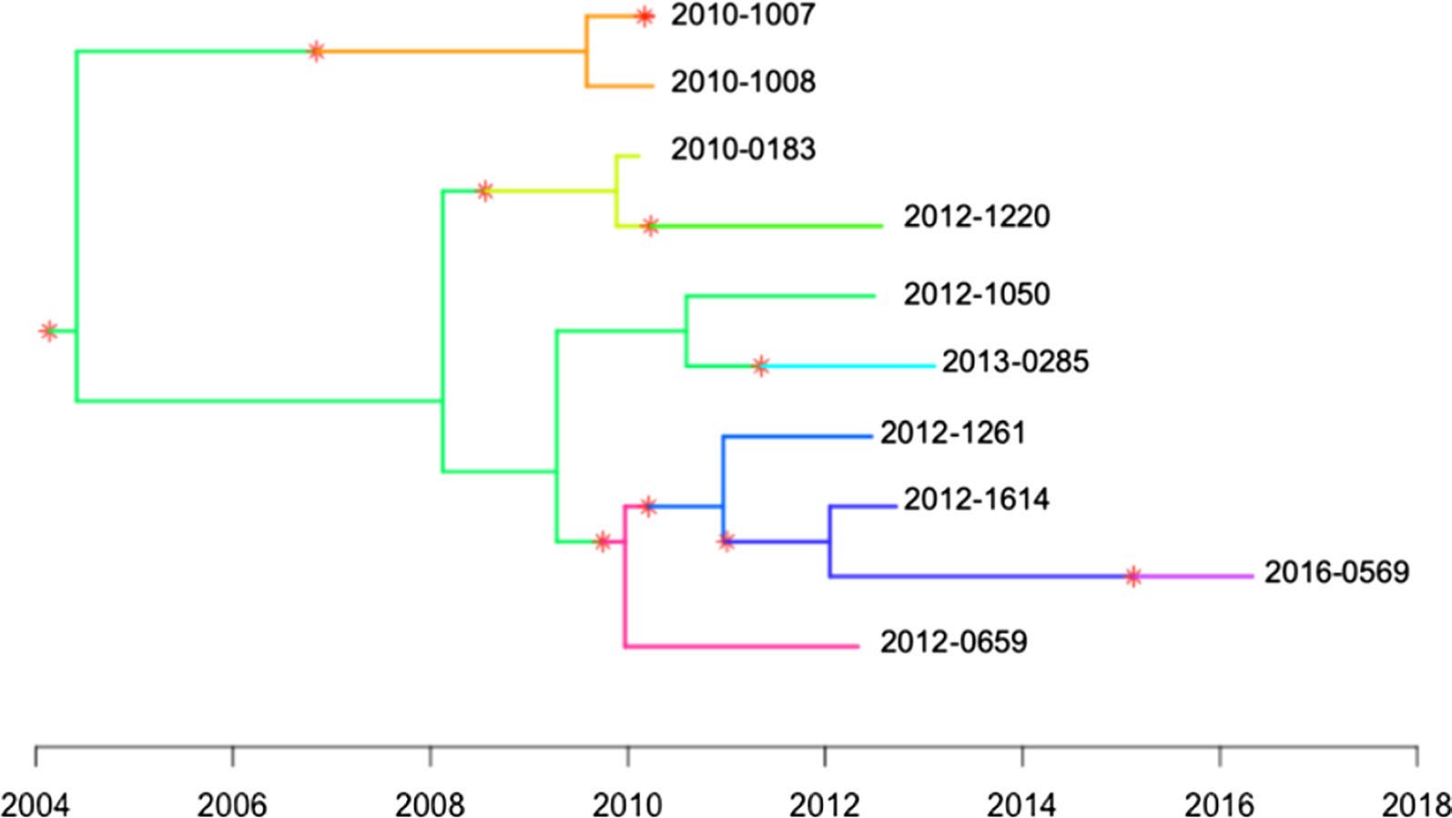
Transmission tree based on the time-labeled phylogenic tree of the ten MDR-TB patients. The star and the change of color represent the occurrence of transmission event or new infection

We combined WGS and field epidemiological investigations from a cluster of ten MDR-TB patients to infer the transmission route. Current evidence and the transmission tree (Figs. 1 and 2) suggest that this cluster initialed as early as 2006, with unsampled source cases. The source case, likely infected before 2006, transmitted the MDR *M. tuberculosis* strain to at least 9 cases in this community. The timed tree (Additional file 1: Figure S3) indicated an unsampled source case for this cluster. Due to the lack of previous strains from these two patients, we cannot rule out the possibility of other source cases. The timed-tree also showed that there were multiple transmission events in this community; however, due to the relocation of residents living in this alley, the transmission of this MDR-TB strain may seed in other communities, and further contact tracing should be reinforced.

In the field epidemiological investigation, we found that these transmission events likely occurred in the household or a commonly frequented game room. There were reports in China that the transmission of TB may be due to accidental exposure [23], which indicated that most patients in a cluster had no apparent history of contact with other patients. Many molecular epidemiological studies abroad reported a lower proportion of cluster cases who had determined contact history [24–26]. Our investigation found that some of the patients in this cluster had occasional contacts in the game room, suggesting that short contacts that do not typically qualify as long-term contacts may lead to infection. In this study, we were impressed that this neighborhood was one of the largest traditional residences in Shanghai, which was eventually demolished in April 2013. Before the demolition, there were 2500 households and about 4000 residents living in the area of 40,000 square meters, and the population density was 1/10 m^2^. There were several commonly frequented, poorly ventilated fast-food restaurants and family game rooms.

As a result of the relocation and other historical reasons, many of the tenants of the alley, who may have been exposed moved away. Given the suggestion that casual contacts may lead to successful transmission, public health officials should take measures to discover and treat MDR-TB patients among those potentially exposed households to control the transmission of MDR-TB. This also raises the limitation that this demolition of the alley could lead to an incomplete contact evaluation.

MDR-TB patients may have a longer extended period of infection due to delayed diagnosis and treatment, and delays in sputum *Mycobacteria* conversion. [27, 28]. The treatment of MDR-TB in China has a relatively low cure or completion rate (< 50%) [28]. In this study of 10 MDR-TB patients, only one patient was cured. Incomplete treatment may facilitate the transmission of MDR-TB. Our findings suggest that a surveillance system integrating molecular genotyping may detect putative transmission of MDR-TB strains in the community and inform the epidemiological investigation of the transmission network. Routine or ideally real-time implementation of the genotyping and WGS tools may be informative to initial the alarm of possible outbreaks. However, due to the technological and financial reasons, the powerful genomic sequencing is not available or at impractical costs in many high TB burden regions including the rural parts of China. Therefore, the WGS cannot be presented as the only solution capable of tracing the epidemiological relationships for the control of tuberculosis in different countries, as it has been systematically emphasized in scientific publications on the molecular characterization of *M. tuberculosis*.

In addition to the transmission of drug-resistant strains, we demonstrate evidence of additional acquisition of drug-resistant mutations. WGS data analysis demonstrated that during the course of transmission, INH, SM, and RIF related resistance mutations were fixed, and EMB, PZA, and FLQ resistance mutations were accumulated gradually in several MDR-TB cases located in the end-branch of the phylogeny tree. The accumulation of additional drug-resistant mutations may result from incomplete or interrupted treatment due to serious treatment adverse effects and/or low treatment success rates. Treatment programs for MDR-TB patients should be individualized and standardized based on (DST) to increase the probability of successful treatment shorten the infectious period. Zhang and colleagues [29] found that adverse factors affecting the treatment effect of MDR-TB patients included long-term treatment and the initial drug-resistant types, suggesting short-term and effective treatment strategies may be crucial to contain the spreading of MDR-TB.

## Conclusions

In summary, we observed a local transmission of MDR-TB in one relocated-community in Jing’an district in Shanghai. Our findings suggest that intensive TB screening might help to control local TB epidemics, especially in settings where there is a local transmission of MDR-TB. In order to impede transmission, public health efforts should increase the intensity of the active case finding of MDR-TB and target interventions to shared spaces that are commonly frequented, even for a short amount of time. This is especially true of poorly ventilated spaces. Additionally, rapid diagnostic technology to screen for TB and drug-resistance may enhance the early detection of MDR-TB. These intensive interventions could improve the monitoring, follow-up, and management of drug resistance TB patients, and may ultimately reduce the transmission of MDR-TB.

## Supplementary Information

**Additional file 1: Figure S1.** Maximum-likelihood tree of ten MDR-TB strains and H37Rv. The bootstrap was showed as percentage of 1000 runs. **Figure S2.** Maximum-likelihood tree of ten MDR-TB strains. The bootstrap was showed as percentage of 1000 runs. **Figure S3. **Estimated transmission tree based on the time-labeled phylogenic tree of the ten MDR-TB patients. The star and the change of color represent the occurrence of transmission event or new infection.

## Data Availability

All data generated or analyzed during this study are included in this published article and its Additional file 1. All the sorted BAM files of the sequences were deposited in the NCBI Bioproject (PRJNA679962).

## Abbreviations

MDR-TB: Multidrug-resistant TB
WGS: Whole-genome sequencing
SNPs: Single nucleotide polymorphisms
VNTR: Variable numbers of tandem repeats
RIF: Rifampicin
INH: Isoniazid
EMB: Ethambutol
SM: Streptomycin
EMB: Ethambutol
PZA: Pyrazinamide
CTAB: Cetyl trimethyl ammonium bromide
MCMC: Markov Chain Monte Carlo
