# Supplementary material for: Transmission of multidrug-resistant tuberculosis in Shimen community in Shanghai, China: a molecular epidemiology study
Source: BMC Infect Dis. 2021 Oct 29;21:1118. doi: 10.1186/s12879-021-06725-0 (PMC8557015; doi:10.1186/s12879-021-06725-0)

Transmission of Multidrug-resistant tuberculosis in Shimen community in Shanghai, China: a molecular epidemiology study

Authors: Zhiying Han[1*], Jing Li[2*], Guomei Sun[1], Kaikan Gu[1], Yangyi Zhang[2], Hui Yao[3], and Yuan Jiang[2]

[1] Department of Tuberculosis Prevention and Control, Jing'an District Center for Disease Control and Prevention, Shanghai,200072, China.

[2] Tuberculosis Laboratory, Shanghai Municipal Center for Disease Control and Prevention, Shanghai, 200036, China.

[3] Second Shimen Road Community Health Center, Shanghai, China

Appendix:

Figure S1. Maximum-likelihood tree of ten MDR-TB strains and H37Rv. The bootstrap was showed as percentage of 1000 runs.

Figure S2. Maximum-likelihood tree of ten MDR-TB strains. The bootstrap was showed as percentage of 1000 runs.

Figure S3. Estimated transmission tree based on the time-labeled phylogenic tree of the ten MDR-TB patients. The star and the change of color represent the occurrence of transmission event or new infection.

Figure S1. Maximum-likelihood tree of ten MDR-TB strains and H37Rv. The bootstrap was showed as percentage of 1000 runs.

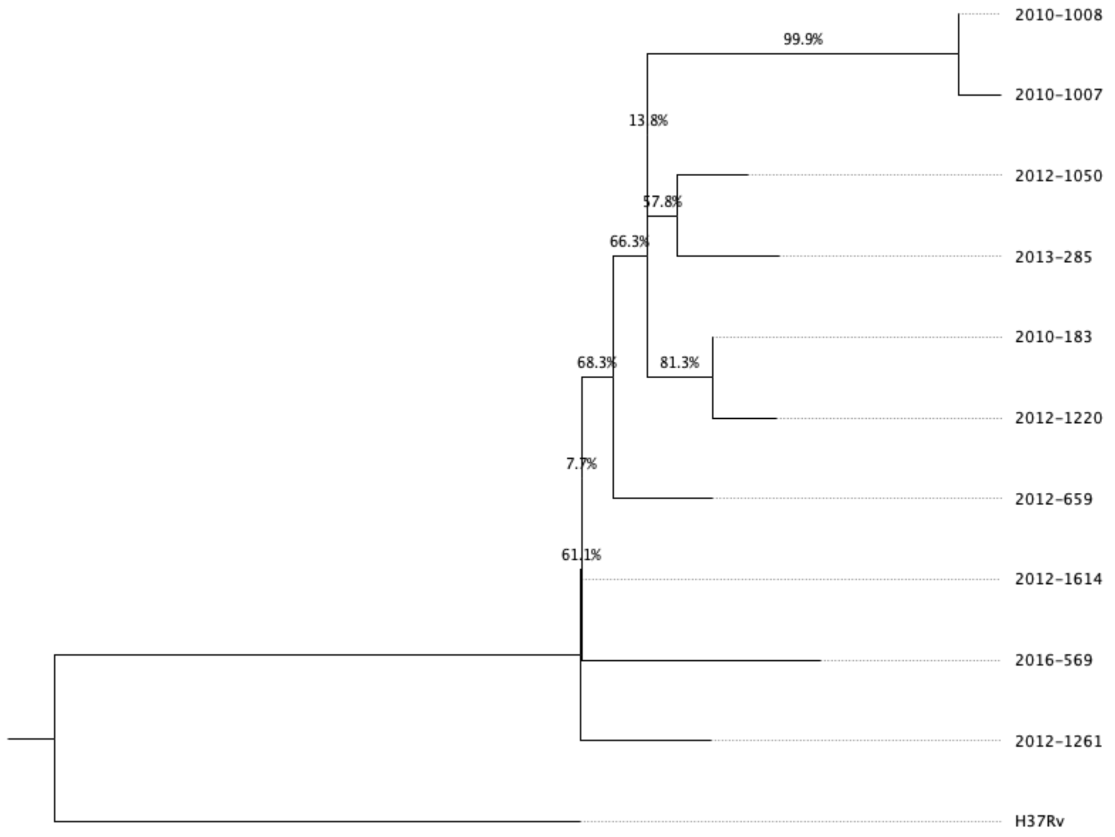

Figure S2. Maximum-likelihood tree of ten MDR-TB strains. The bootstrap was showed as percentage of 1000 runs.

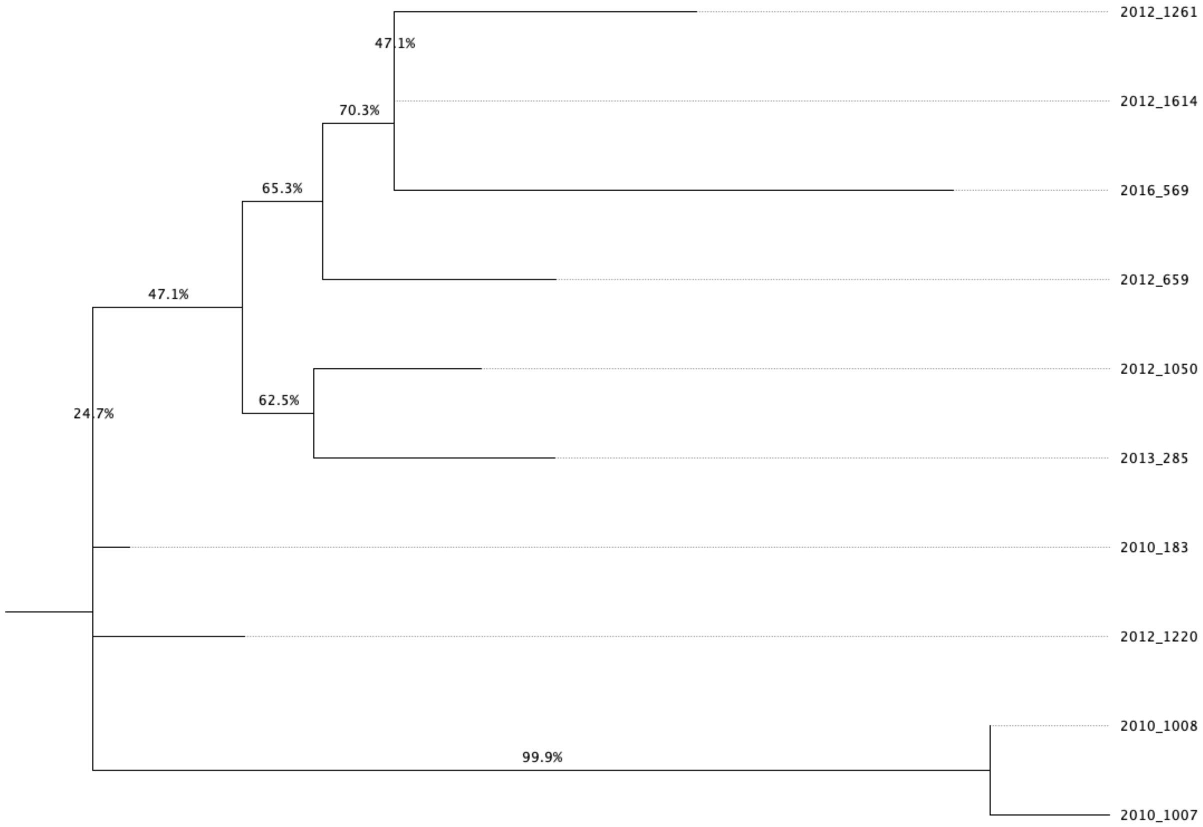

Figure S3. Estimated transmission tree based on the time-labeled phylogenic tree of the ten MDR-TB patients. The star and the change of color represent the occurrence of transmission event or new infection.

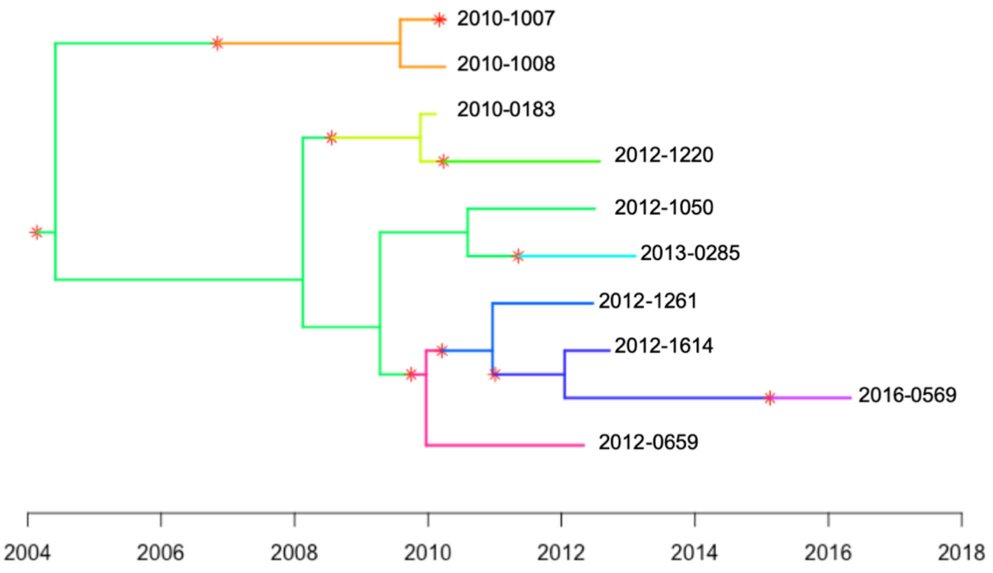

Supplement: Supplementary file 1 — Additional file 1: Figure S1. Maximum-likelihood tree of ten MDR-TB strains and H37Rv. The bootstrap was showed as percentage of 1000 runs. Figure S2. Maximum-likelihood tree of ten MDR-TB strains. The bootstrap was showed as percentage of 1000 runs. Figure S3. Estimated transmission tree based on the time-labeled phylogenic tree of the ten MDR-TB patients. The star and the change of color represent the occurrence of transmission event or new infection. [file 12879_2021_6725_MOESM1_ESM.pdf]
